# Sclerosing angiomatoid nodular transformation of the spleen in an 18-year-old man: a case report

**DOI:** 10.1093/jscr/rjag473

**Published:** 2026-09-16

**Authors:** Keisuke Yamazaki, Shinji Tsutsumi, Amane Hamamoto, Hinako Kikuchi, Shunsuke Kubota, Harue Akasaka, Yoshiyuki Sakamoto, Shigeru Shibata

**Affiliations:** Department of Gastroenterological Surgery, Hirosaki General Medical Center, 1 Tomino-cho, Hirosaki, Aomori, 036-8545, Japan; Department of Gastroenterological Surgery, Hirosaki General Medical Center, 1 Tomino-cho, Hirosaki, Aomori, 036-8545, Japan; Department of Gastroenterological Surgery, Hirosaki General Medical Center, 1 Tomino-cho, Hirosaki, Aomori, 036-8545, Japan; Department of Gastroenterological Surgery, Hirosaki General Medical Center, 1 Tomino-cho, Hirosaki, Aomori, 036-8545, Japan; Department of Gastroenterological Surgery, Hirosaki General Medical Center, 1 Tomino-cho, Hirosaki, Aomori, 036-8545, Japan; Department of Gastroenterological Surgery, Hirosaki General Medical Center, 1 Tomino-cho, Hirosaki, Aomori, 036-8545, Japan; Department of Gastroenterological Surgery, Hirosaki General Medical Center, 1 Tomino-cho, Hirosaki, Aomori, 036-8545, Japan; Department of Gastroenterological Surgery, Hirosaki General Medical Center, 1 Tomino-cho, Hirosaki, Aomori, 036-8545, Japan

**Keywords:** sclerosing angiomatoid nodular transformation, spleen, splenic tumor, laparoscopic splenectomy

## Abstract

Sclerosing angiomatoid nodular transformation (SANT) is a rare benign vascular lesion of the spleen, particularly uncommon in adolescents. We report the case of an 18-year-old man with an incidentally detected splenic mass showing a characteristic spoke-wheel enhancement pattern. Although a benign lesion was suspected, malignancy could not be excluded, and laparoscopic splenectomy was performed. Histopathological examination confirmed SANT, and the postoperative course was uneventful. A review of previously reported cases in patients younger than 20 years revealed a median age of 8.5 years, with abdominal pain in 76.9% and nonspecific laboratory abnormalities in 45.5% of cases. Partial splenectomy was performed in 46.7% of patients; however, tumors located at the splenic hilum were consistently treated with total splenectomy. SANT in adolescents is rare and difficult to diagnose preoperatively. Tumor location, particularly when located at the splenic hilum, may be a key factor in determining surgical strategy.

## Introduction

Sclerosing angiomatoid nodular transformation (SANT) is a rare benign vascular lesion of the spleen first described by Martel *et al*. [1] in 2004. It has most commonly been reported in middle-aged adults and is often detected incidentally during imaging studies. Occurrence in pediatric and adolescent populations is extremely uncommon.

Radiologically, SANT may demonstrate a characteristic spoke-wheel enhancement pattern on contrast-enhanced imaging; however, this finding is not specific, and differentiation from malignant splenic tumors often requires histopathological confirmation. We report a rare case of SANT in an 18-year-old man treated with laparoscopic splenectomy and present a review of previously reported cases in patients younger than 20 years.

## Case report

An 18-year-old man with no significant medical history was referred to our hospital after an abnormal shadow was detected on a routine chest radiograph. Subsequent chest computed tomography (CT) incidentally revealed a splenic mass. The patient had no abdominal symptoms. Physical examination revealed no palpable abdominal mass, and laboratory tests, including inflammatory and tumor markers, were within normal limits.

**Figure 1:**
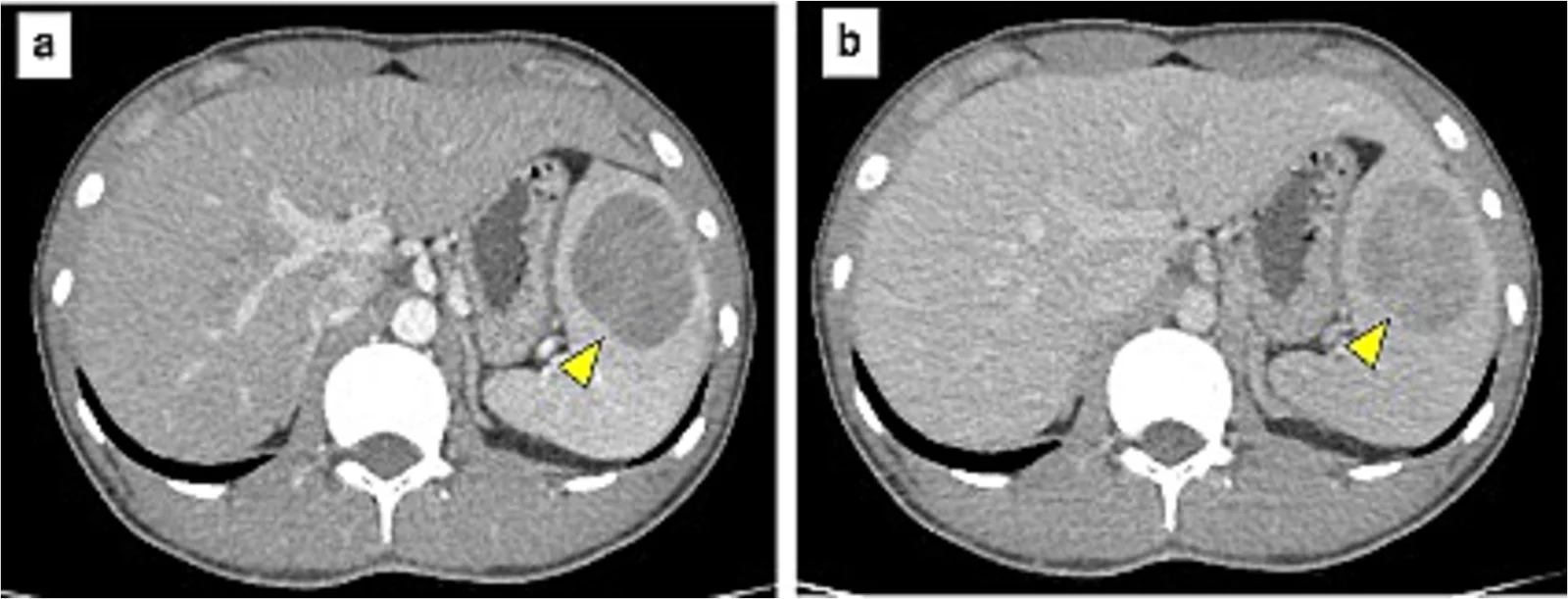
Contrast-enhanced CT. Arterial phase (a) and equilibrium phase (b) images show a well-defined splenic mass with a characteristic spoke-wheel–like enhancement pattern. In the arterial phase, radial linear enhancement is observed from the periphery toward the center of the lesion. In the equilibrium phase, the enhancement progresses centrally.

**Figure 2:**
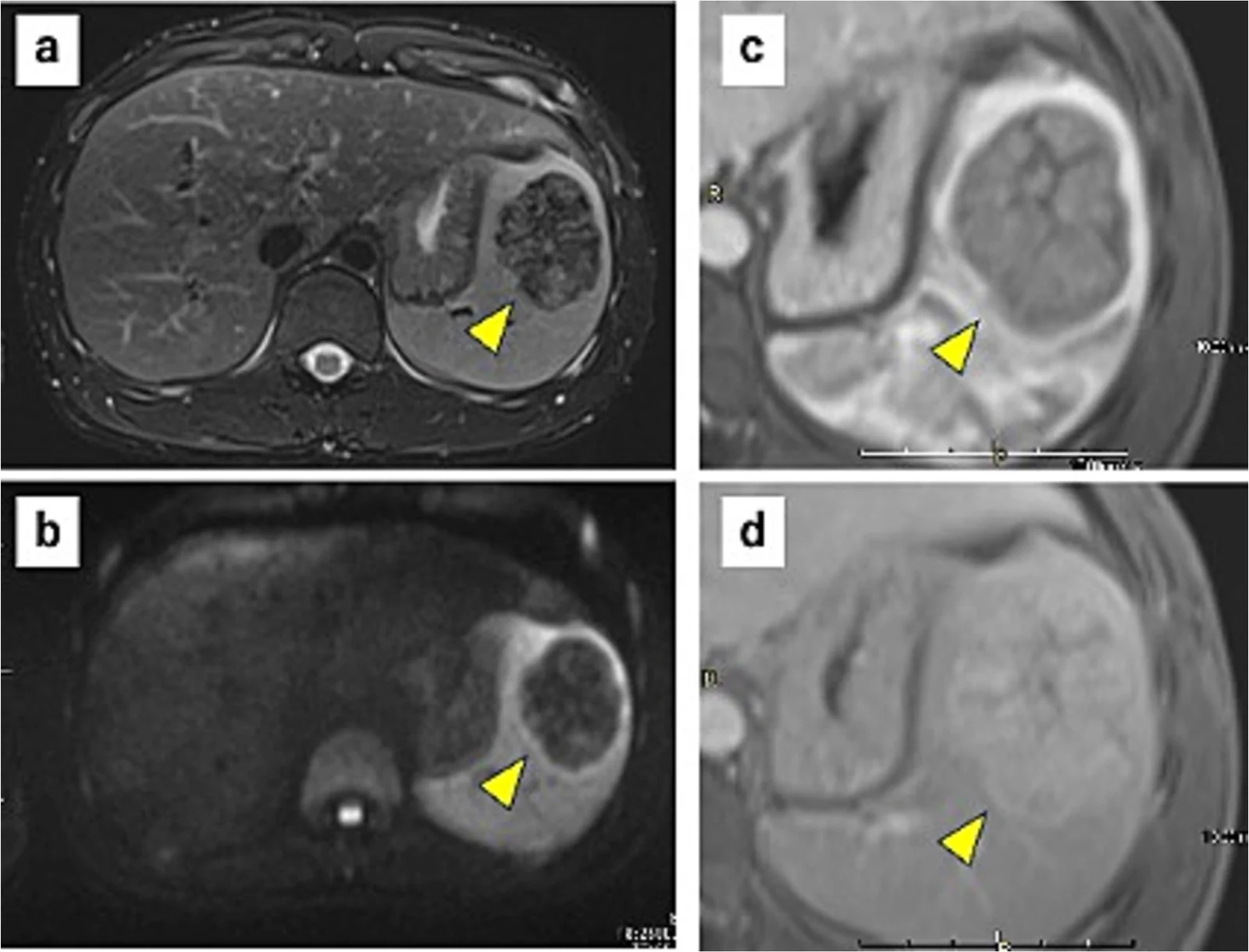
MRI. (a) T2-weighted image showing heterogeneous low-to-isointense signal intensity. (b) Diffusion-weighted image demonstrating low signal intensity. (c) Arterial phase and (d) equilibrium phase contrast-enhanced MRI images show a spoke-wheel–like enhancement pattern similar to that observed on CT.

**Figure 3:**
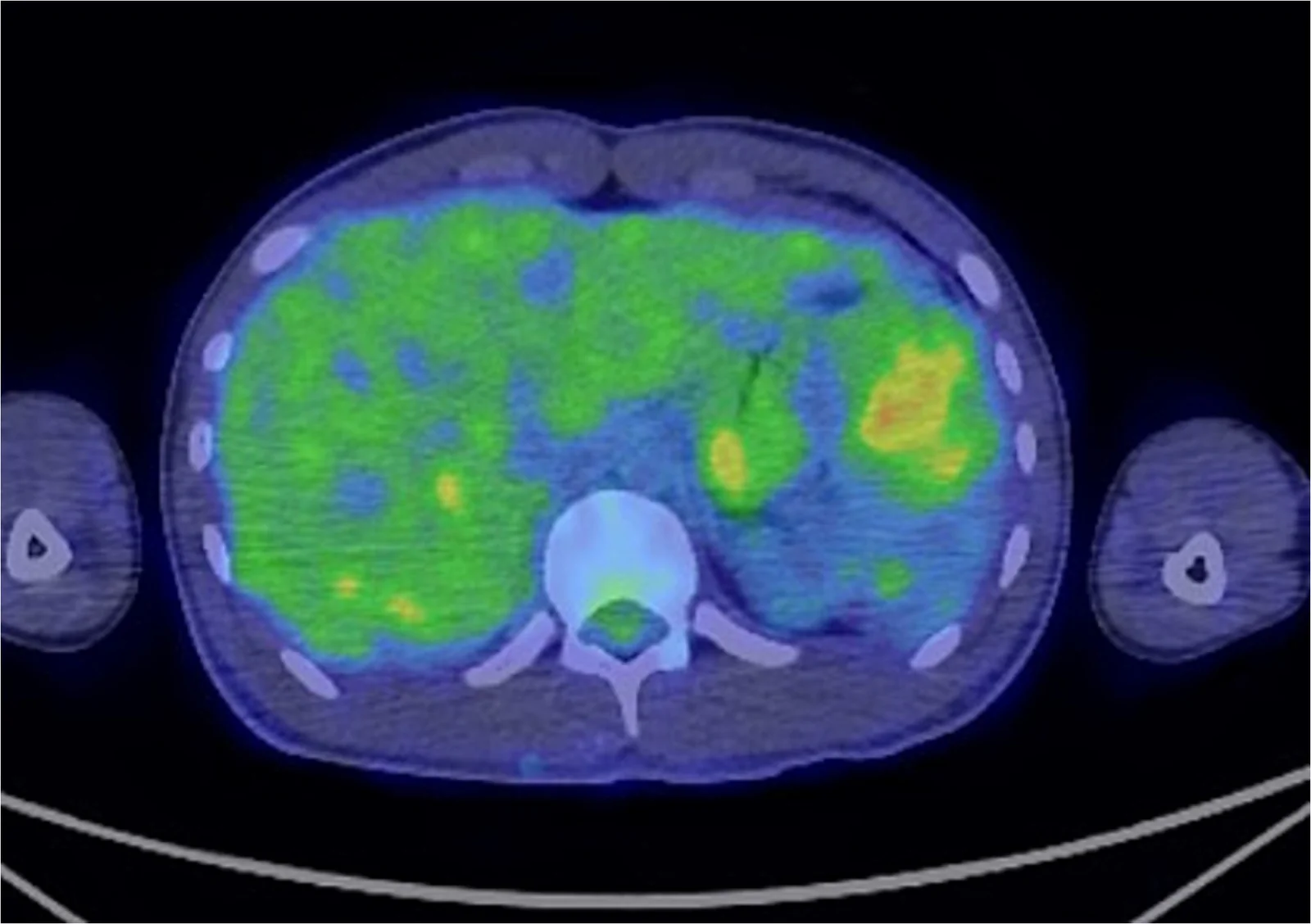
Positron emission tomography-computed tomography (PET-CT). PET-CT shows mild fluorodeoxyglucose uptake within the splenic lesion, with a maximum standardized uptake value (SUVmax) of 3.0. No intense accumulation suggestive of malignancy is observed.

Contrast-enhanced CT demonstrated a solitary, well-defined splenic mass measuring approximately 5 cm, extending from the upper pole to the splenic hilum. In the arterial phase, radial enhancement from the periphery toward the center produced a spoke-wheel-like pattern (Fig. 1). Magnetic resonance imaging (MRI) showed heterogeneous low-to-isointensity on T2-weighted imaging and similar enhancement patterns after contrast administration (Fig. 2). Fluorodeoxyglucose positron emission tomography demonstrated mild uptake with a maximum standardized uptake value (SUVmax) of 3.0 (Fig. 3). Based on the characteristic enhancement pattern, SANT was considered the most likely diagnosis, with inflammatory tumor and hamartoma included in the differential diagnosis. However, malignant splenic tumors, particularly angiosarcoma, could not be excluded preoperatively.

As the tumor extended to the splenic hilum and the vascular anatomy appeared complex, partial splenectomy was considered technically difficult. Laparoscopic total splenectomy was therefore performed after preoperative pneumococcal vaccination.

**Figure 4:**
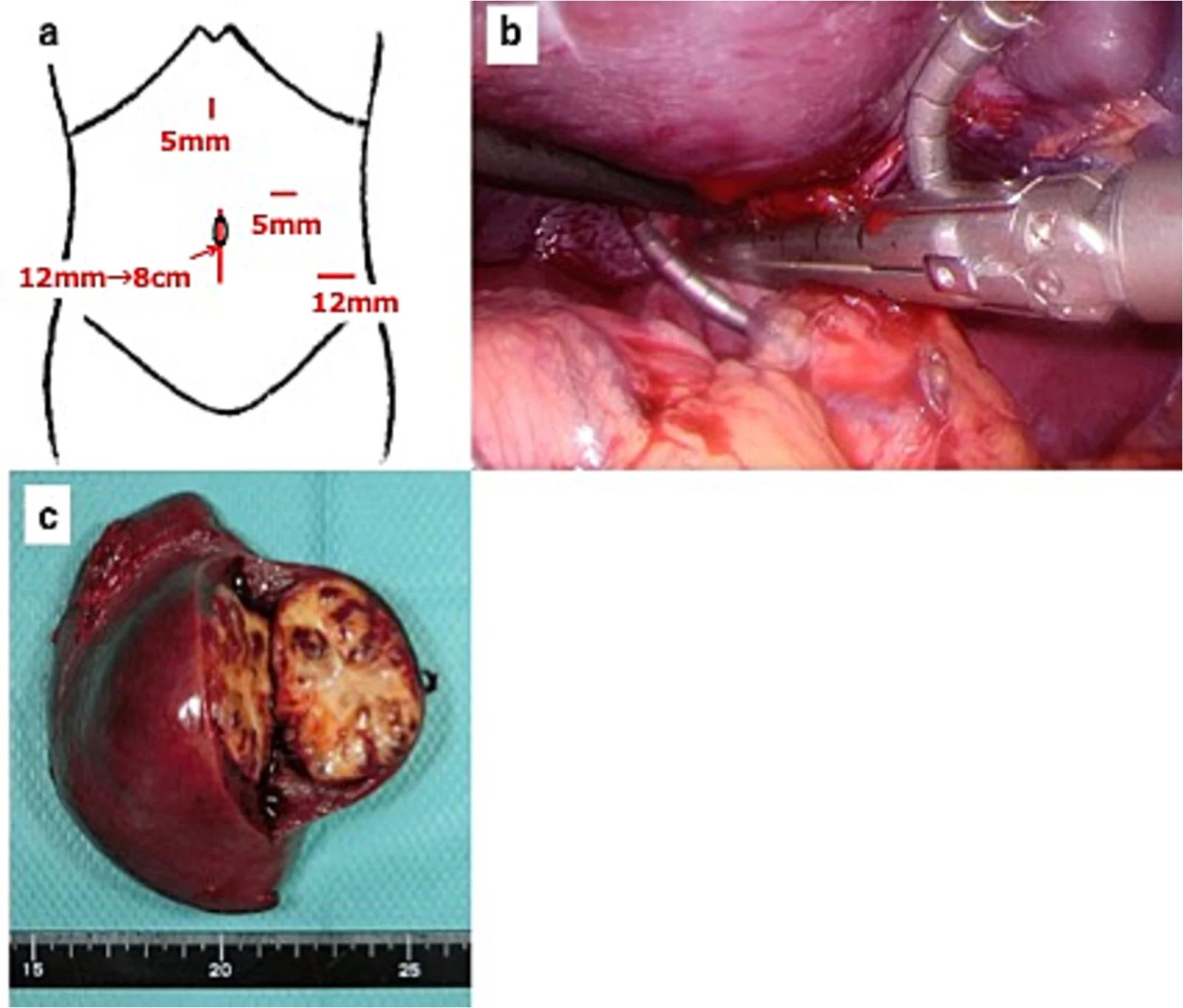
Intraoperative findings and gross specimen. (a) Laparoscopic view showing the surgical setup with four ports. (b) Dissection around the splenic hilum with division of hilar vessels using a laparoscopic linear stapler. (c) Gross specimen showing a well-demarcated nodular lesion extending from the upper pole to the splenic hilum.

The operative time was 157 min with minimal blood loss (<5 mL) (Fig. 4). The postoperative course was uneventful, and the patient was discharged on postoperative day 7.

**Figure 5:**
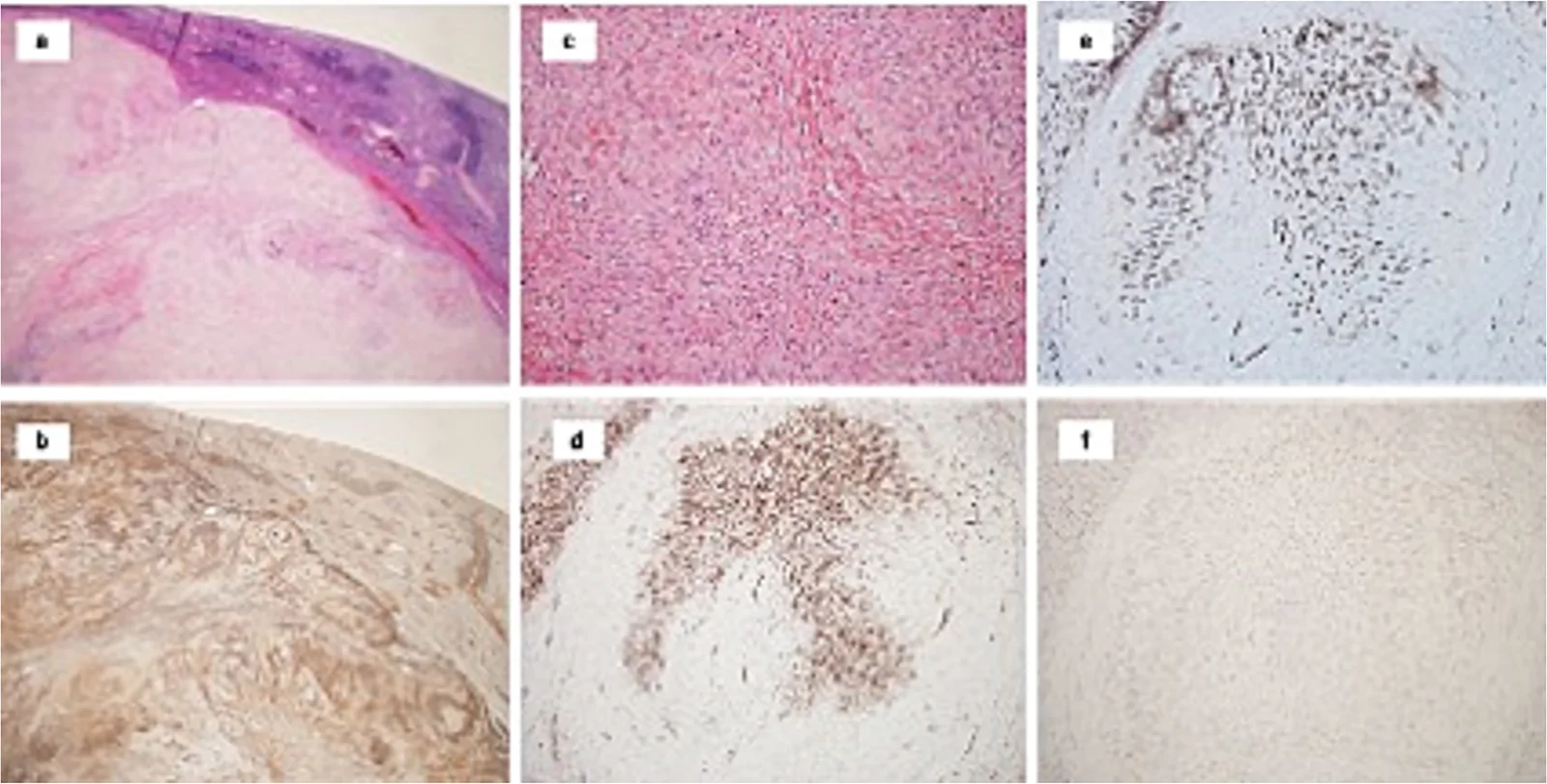
Histopathological and immunohistochemical findings. (a) Hematoxylin-eosin (HE) staining (low magnification) showing a well-circumscribed nodular lesion. (b) α-smooth muscle actin staining showing abundant spindle-shaped fibroblasts. (c) HE staining (high magnification) demonstrating fibrosclerotic nodules. (d) CD31 immunostaining highlighting vascular structures. (e) CD34 immunostaining showing endothelial cell positivity. (f) CD68 immunostaining revealing scattered macrophages.

Histopathological examination demonstrated multiple angiomatoid nodules separated by fibrosclerotic stroma. Immunohistochemical staining showed CD31- and CD34-positive vascular structures and scattered CD68-positive macrophages, confirming the diagnosis of SANT (Fig. 5).

The patient remained well without recurrence, infection, or thrombotic complications during 12 months of follow-up.

## Discussion

SANT is a rare benign vascular lesion of the spleen, predominantly reported in middle-aged adults, with pediatric and adolescent cases being uncommon [1].

In previously reported cases of SANT in patients younger than 20 years [2–13], the median age was 8.5 years (range, 0–18 years), with no apparent sex predominance. Clinical presentation was variable; although some cases were incidentally detected, abdominal pain was observed in approximately 76.9% of patients, indicating that SANT in younger individuals is not always asymptomatic. Laboratory findings were nonspecific, with abnormalities such as anemia reported in approximately 45.5% of cases (Table 1).

**Table 1:** Clinical characteristics and surgical outcomes of reported SANT cases in patients younger than 20 years.

No.	Author	Age (years)	Sex	Symptoms	Laboratory abnormalities	Location (H or U/L)	Tumor size (cm)	Preoperative diagnosis	Treatment (Total/Partial splenectomy)	Postoperative complications	Recurrence
1	Ramezani F, *et al*.	12	M	Yes	No	U/L	4.7	Hemangioma, hamartoma, fibroma, or inflammatory pseudotumor	Partial	None	None
2	Kim JY, *et al*.	8	M	Yes	Yes	U/L	4.8	SANT suspected; atypical hemangioma or inflammatory pseudotumor	Partial	None	None
3	Xia L, et al.	15	F	No	No	U/L	9.7	Unknown	Total	None	None
4	Soleimani N, *et al*.	3	F	Yes	Yes	U/L	5	Lymphoma suspected	Partial	None	None
5	Wang Z, *et al*.	10	F	Yes	-	U/L	5.5	Unknown	Partial	-	-
6	Wang Z, *et al*.	6	F	Yes	-	H	4.5	Unknown	Total	-	-
7	Wang Z, *et al*.	9	M	Yes	-	U/L	4.5	Unknown	Total	-	-
8	Jamal A, *et al*.	8	F	-	Yes	-	-	Unknown	Total	None	None
9	Idrissa S, *et al*.	14	F	No	Yes	H	7	Unknown	Total	None	None
10	Idrissa S, *et al*.	4	M	Yes	No	U/L	3	Unknown	Partial	None	None
11	Pelizzo G, *et al*.	0	F	Yes	Yes	U/L	-	Intra-abdominal hemorrhage from a splenic lesion	Total	None	None
12	Cao P, *et al*.	7	M	-	-	-	9.5	Hamartoma	-	None	None
13	Zhang S, *et al*.	3	M	Yes	No	U/L	5	Hamartoma, hemangioma, or low-grade lymphoma	Partial	None	None
14	Vyas M, *et al*.	11	M	Yes	No	-	5	Unknown	Partial	None	None
15	Kuybulu A, *et al*.	11	F	-	-	-	-	Unknown	Total	None	None
16	Our case	18	M	No	No	H	5.1	SANT suspected; angiosarcoma could not be excluded	Total	None	None

OPSI: overwhelming post-splenectomy infection

tumor location was categorized as splenic hilum or upper/lower pole.

Treatment was classified as total splenectomy or partial splenectomy.

No cases of postoperative OPSI, thrombotic complications, or recurrence were reported among the reviewed cases.

The differential diagnosis of SANT includes benign vascular lesions such as hemangioma, hamartoma, and littoral cell angioma; inflammatory pseudotumor/inflammatory myofibroblastic tumor; and malignant lesions such as lymphoma, angiosarcoma, and splenic metastasis. The spoke-wheel-like enhancement pattern, progressive centripetal enhancement, and low-signal fibrous bands on T2-weighted MRI are suggestive of SANT but are not specific. Hemangioma and hamartoma may show overlapping enhancement patterns, whereas lymphoma is often associated with splenomegaly and/or lymphadenopathy. Angiosarcoma may show heterogeneous enhancement with necrosis, hemorrhage, or distant metastasis, and metastatic splenic tumors should be considered in patients with a history of malignancy. In the present case, although SANT was included in the differential diagnosis based on imaging findings, malignant splenic tumors such as lymphoma, angiosarcoma, and metastasis could not be excluded preoperatively [14, 15].

Our review suggests that tumor location plays a critical role in determining surgical strategy. In previously reported cases, partial splenectomy was performed in 46.7% of patients, indicating that spleen-preserving surgery is feasible in selected cases (Table 1). However, lesions located at the splenic hilum were consistently treated with total splenectomy, whereas lesions located in the upper or lower pole were more frequently managed with partial splenectomy. This likely reflects the anatomical complexity of the splenic hilum and the technical difficulty of preserving vascular supply. In our case, the tumor extended to the splenic hilum, making partial splenectomy impractical, and laparoscopic total splenectomy was considered the safest approach.

Importantly, no cases of overwhelming post-splenectomy infection, thrombotic complications, or recurrence were reported among the reviewed cases, suggesting that surgical outcomes for pediatric and adolescent SANT are generally favorable regardless of the surgical approach.

This report has some limitations. The number of available cases remains limited, and the analysis is based on previously reported cases, which may introduce reporting bias.

In conclusion, SANT in adolescents is rare and difficult to distinguish from malignant tumors preoperatively. Tumor location, particularly when located at the splenic hilum, appears to be a key factor in determining the feasibility of spleen-preserving surgery.
